# Psychometric properties of the Brazilian 14-Item Health Literacy Scale for people living with mental disorders: a cross-sectional study

**DOI:** 10.1590/1806-9282.20251444

**Published:** 2026-10-12

**Authors:** Marcos Renan Barbosa, Lucas Machado Maracci, Marília Jesus Batista de Brito Mota, Eliana Elisa Rehfeld Gheno, Márcio Strassburger, Mariana Fröhlich Alievi, Olga Maria Martins de Sousa Valentim, Adriane Cristina Bernat Kolankiewicz

**Affiliations:** 1 Universidade Regional do Noroeste do Estado do Rio Grande do Sul, Graduate Program in Comprehensive Health Care – Ijuí (RS), Brazil.; 2 Universidade Federal de Santa Maria, Graduate Program in Dental Sciences – Santa Maria (RS), Brazil.; 3 Faculdade de Ciências Médicas da Santa Casa de São Paulo, Graduate Program in Dentistry – São Paulo (SP), Brazil.; 4 University of Lisbon, School of Nursing, Nursing Research Innovation and Development Centre of Lisbon (CIDNUR) – Lisbon, Portugal.; 5 RISE-Health, Nursing School of Porto – Porto, Portugal.

**Keywords:** Health literacy, Mental disorders, Validation study, Brazil, Mental health

## Abstract

**INTRODUCTION:**

There is no evidence of validity and reliability for an instrument capable of comprehensively assessing health literacy (including its Functional, Communicative, and Critical dimensions) among Brazilian adults living with mental disorders.

**OBJECTIVE:**

The aim of this study was to evaluate the validity, reliability, and evidence of divergent validity of the Brazilian 14-Item Health Literacy Scale in individuals with chronic mental disorders.

**METHODS:**

This cross-sectional psychometric study involved patients receiving care at the Psychosocial Care Center in the city of Ijuí, southern Brazil, between April and October 2023. Eligible participants were clinically stable adults with severe and persistent mental disorders; individuals with intellectual disabilities or legal incapacity were excluded. Data collection was conducted by trained students, who read and explained the items of each instrument to the participants. In total, the sample comprised 444 patients. The Brazilian version of the 14-Item Health Literacy Scale, previously translated, cross-culturally adapted, and validated for Brazilian Portuguese, was used to assess health literacy and to perform the psychometric analysis.

**RESULTS:**

Good internal consistency was demonstrated, with Cronbach's alpha and McDonald's omega coefficients of 0.83 (95%CI 0.78–0.88) and 0.75 (95%CI 0.68–0.83), respectively. The 14-Item Health Literacy Scale presented acceptable fit indices for the Comparative Fit Index, Tucker-Lewis Index, and Normed Fit Index, with values of 0.938, 0.923, and 0.904, respectively. Divergent validity was verified by the lack of statistically significant correlations and by weak correlations (r<0.300) with the Social Support Scale.

**CONCLUSION:**

The 14-Item Health Literacy Scale presented adequate psychometric properties and showed evidence of validity and acceptable reliability for assessing the health literacy of Brazilians living with mental disorder.

## INTRODUCTION

Mental disorders (MDs) are syndromes resulting from psychological, biological, or developmental dysfunctions that impair cognition, emotions, and behavior^1^. The prevalence of common MDs in Brazil is approximately 20%^2^, and an estimated 5 million individuals have severe and persistent MDs^3^. Studies conducted in Psychosocial Care Centers (CAPS) indicate a higher frequency of schizophrenic, schizotypal, and delusional disorders^4^. Due to their chronicity, associated disabilities, and socioeconomic impact, MDs represent a significant public health issue^5^, affecting individuals, families, and society^6^. Furthermore, due to the chronicity and disability caused by MDs and the consequent impact on the individual and collective economy, these disorders have become an important public health issue to be discussed^7^.

Health literacy (HL) refers to the ability to obtain, understand, and use health information^8^, in order to make decisions and take actions that promote one's own well-being and that of others, as well as the ability to identify and implement actions aimed at promoting health and improving quality of life. Higher HL levels are associated with better autonomy and treatment adherence^9-11^, whereas low HL impairs health management and is associated with lower utilization of health services and greater difficulty in autonomously managing health conditions^12,13^.

HL comprises three dimensions: Functional, Communicative, and Critical. The Functional dimension refers to basic reading and writing skills used in everyday situations; the Communicative dimension involves more advanced cognitive and social skills; lastly, the Critical dimension refers to a set of cognitive and social skills that enable individuals to critically analyze information and apply it to situations in their daily lives^14^. There are several instruments in the literature that seek to assess Functional HL^15^. However, few instruments are capable of assessing the different dimensions of HL, such as the 14-Item Health Literacy Scale (HLS-14)^16^, which is an instrument validated for use in adults regardless of health status. In 2020, it was validated and translated into Brazilian Portuguese after application to a cohort of Brazilian adults, and demonstrated good internal consistency^17^.

In Brazil, HL among people with MDs remains understudied^18,19^, as there is no validated instrument available to measure this construct in adults living with this condition, which limits both research and the assessment of this important variable in this population. Furthermore, most studies have focused exclusively on the assessment of functional HL^15,20^. Given the high prevalence of MDs^2^ and the need for a valid and reliable instrument to assess HL from the perspective of people living with MDs, this study aimed to evaluate the validity, reliability, and evidence of divergent validity of the Brazilian HLS-14 in individuals with chronic mental disorders.

## METHODS

The study was approved by the Research Ethics Committee of the Regional University of Northwest Rio Grande do Sul (protocol no. 5.966.864/2022), in accordance with the 1964 Helsinki Declaration and its subsequent amendments.

This is a cross-sectional psychometric study conducted at a Psychosocial Care Center (CAPS) located in the city of Ijuí, southern Brazil, that has 83,947 inhabitants, integrated into the matrix project "Health Literacy from the Perspective of People with Mental Disorders and Health Professionals: A Mixed-Methods Study." Participants were users with severe and persistent mental disorders in stable clinical condition. Individuals with intellectual disability/mental retardation, judicial interdiction, and/or International Classification of Diseases, 10th Revision (ICD-10) diagnoses F70–F79 were excluded.

Data collection occurred between April and October 2023, conducted by previously trained students. Sample size calculation^21^ considered a 90% confidence level and a 9.5% margin of error for a population of 720 users, resulting in a minimum of 432 participants. Participants were recruited through convenience sampling until the target sample size was achieved. Eligibility was assessed according to the inclusion and exclusion criteria. Upon arriving at the facility, patients were invited to participate in the study. After agreeing to participate, they signed the Informed Consent Form and were then escorted to a private room to ensure confidentiality and privacy.

The following instruments were applied: a sociodemographic questionnaire; the HLS-14^17^; and the Medical Outcomes Study Social Support Scale (MOS-SSS)^22^. The HLS-14 is a validated instrument for assessing HL in Brazil^17^; it comprises 14 items (5 assessing Functional HL, 5 assessing Communicative HL, and 4 assessing Critical HL) rated on a five-point Likert scale that indicates how much the participant agrees or disagrees with the item ("strongly disagree" to "strongly agree"). For items 1–5 (Functional dimension), scores range from 1 to 5, with agreement indicating lower HL; in contrast, for items 6–10 (Communicative dimension) and 11–14 (Critical dimension), scoring is reversed (5 to 1), as agreement is associated with higher HL. The MOS-SSS is a multidimensional instrument used to assess users’ perceived social support (SS), consisting of 19 items distributed across five dimensions: tangible support, affectionate support, emotional support, informational support, and positive social interaction. Responses are rated on a five-point frequency scale reflecting perceived availability of support: always (5), most of the time (4), sometimes (3), rarely (2), and never (1)^22^.

Statistical analyses were performed using Statistical Package for the Social Sciences (SPSS) 25.0 and Analysis of Moment Structures (AMOS), through Confirmatory Factor Analysis (CFA) within Structural Equation Modeling. Outliers (Mahalanobis distance), normality (skewness, kurtosis, and Mardia's coefficient), maximum likelihood estimation, and model fit indices (χ^2^, Root Mean Square Residual [RMR], Root Mean Square Error of Approximation [RMSEA], Goodness-of-Fit Index [GFI], Comparative Fit Index [CFI], Normed Fit Index [NFI], and Tucker-Lewis Index [TLI]) were assessed. Reliability was evaluated using Cronbach's alpha and McDonald's omega (0.70–0.95). Convergent/divergent validity evidence was examined through linear associations between HLS-14 and MOS-SSS.

## RESULTS

In total, 512 eligible individuals were invited to participate, 68 (13%) of whom declined, resulting in a final sample of 444 participants (87% response rate). Of these, 75.9% were women, 76.5% were white, and 31.8% were between 55 and 64 years old. Most had a household income of up to two minimum wages (77.4%) and lived with others—including partners, children, or other people (83.3%). Medical records revealed diagnoses of depressive disorders (61%), anxiety disorders (15%), bipolar mood disorder (10%), schizophrenia, schizotypal disorders, delusional disorders (11%), personality and behavioral disorders (1%), and other disorders (1%).

Regarding the validation of the HLS-14, the 14 items were distributed across three dimensions. The validation of the scale structure, performed using CFA, initially considered the basic assumptions for applying the technique and identified evidence of univariate normality, with absolute values of skewness and kurtosis estimated below 3 and 5, respectively. However, the Mardia coefficient (Coefficient=56.473) indicated an asymmetric multivariate distribution. Given this limitation, the structural model was estimated using the Maximum Likelihood method.

According to the results obtained, based on the weight of the individual item loadings, the 14 items of the scale saturated with a magnitude greater than 0.400 (p<0.001). The items with the highest loadings are Q8 (λ=0.806), Q7 (λ=0.762), and Q13 (λ=0.765), while items Q10 (λ=0.443); Q6 (λ=0.539); and Q11 (λ=0.568) were items with less weight and less explanatory power, although significant.

Regarding correlations between dimensions, low estimates were detected for the Functional dimension compared to the Communicative (r=-0.340) and Critical (r=-0.250) dimensions. The results with negative coefficients are noteworthy, as the Functional dimension presents reversed items in its assessment. However, between the Communicative and Critical dimensions, the correlation was positive and strong (r=0.710), indicating a degree of dependence between the items. This finding suggests the possibility of these items merging into a single factor, as supported by both the model's quality indices and the dimensions’ internal consistency estimates.

Regarding the results achieved by the three-dimensional model structure, quality indicators (or modification indices) were observed, which allow us to identify how well the sample of 444 individuals conformed to the instrument's original structure. Table 1 presents the goodness-of-fit indices for the three-dimensional model defined by CFA on the HLS-14.

**Table 1:** Goodness-of-fit indices obtained from the confirmatory factor analysis of the original three-factor structure of the Brazilian 14-Item Health Literacy Scale in a sample of adults with mental disorders (n=444).

Data adequacy indices		Confirmatory factor model of the HLS-14
		Estimators
Normed χ^2^ (χ^2^/df)		**2.626**
RMSEA [95%CI]		**0.061 [0.050–0.071]**
Fit indices: comparative indices		
	AGFI	**0.917**
	GFI	**0.941**
	CFI	**0.938**
	TLI	**0.923**
	NFI	**0.904**
Predictive fit indices		
	AIC	**2.006**
	RMR (SRMR)	**0.056 (0.043)**

AGFI: Adjusted Goodness-of-Fit Index; AIC: Akaike Information Criterion; CFI: Comparative Fit Index; GFI: Goodness-of-Fit Index; df: degrees of freedom; CI: confidence interval; HLS-14: 14-Item Health Literacy Scale; NFI: Normed Fit Index; RMR: Root Mean Square Residual; RMSEA: Root Mean Square Error of Approximation; SRMR: Standardized Root Mean Square Residual; TLI: Tucker-Lewis Index; **χ**2: chi-square; **χ**2/df: normed chi-square. The bold values indicate the estimated goodness-of-fit indices obtained from the confirmatory factor analysis.

An initial assessment of the model's parsimonious fit to the sample (χ^2^/df) indicated minimal discrepancy between the observed and estimated covariance matrices, with a goodness-of-fit value below 4.00 ([χ^2^/df]=2.626<4.0). Furthermore, regarding the sample's fit to the scale's theoretical structure, we investigated whether the degree of disagreement between the model and the sample covariance was within the acceptance limits of an adequate fit, using the RMSEA. Also, according to the result obtained (RMSEA=0.061<0.08), the observed disagreements did not compromise the credibility of the model. Comparative indices (CFI=0.938; TLI=0.923; NFI=0.904) indicated satisfactory fit.

As shown in Table 1, the absolute fit indices GFI (Goodness-of-Fit Index) and AGFI (Adjusted Goodness-of-Fit Index) presented values above the minimum acceptable threshold (0.900).

Regarding the relative indexes, which assess how much better the proposed model fits the data compared to the base model generated by AMOS (hypothetical model), the estimates for TLI and CFI were above the acceptable cutoff point, with estimates of 0.923 and 0.938, respectively. The NFI, which investigates the proportion of the difference between the Chi-square value of the proposed model in relation to a null model, was estimated at 0.904 (>0.900), indicating that, for the sample studied, the model presents representative correlations that enable the proposed dimensions.

As for reliability, the Omega (ω) coefficient for the total HLS-14 was 0.755 (95%CI 0.678–0.831), indicating satisfactory reliability (ω>0.700), as shown in Table 2. Regarding the Cronbach's alpha estimate, this coefficient reached 0.831 (95%CI 0.781–0.880). It was observed that the participants presented low Functional literacy (2.83) and high Critical literacy (3.62) and Communicative literacy (3.57).

**Table 2:** Measures of central tendency, variability, and internal consistency (McDonald's omega and Cronbach's alpha) for the dimensions of the Brazilian 14-Item Health Literacy Scale in adults with mental disorders (n=444).

Dimensions	HLS-14 estimates						
	Mean	Standard deviation	Quartiles			Reliability (95%CI)	
			Q1	Median Q2	Q3	ωʄ	αC
Functional HL	2.83	0.86	2.20	2.80	3.40	0.814 (0.740–0.887)	0.826 (0.784–0.867)
Communicative HL	3.57	0.61	3.20	3.60	4.00	0.892 (0.838–0.945)	0.899 (0.845–0.952)
Critical HL	3.62	0.66	3.25	3.75	4.00	0.720 (0.714–0.725)	0.767 (0.700–0.833)
Total	3.32	0.53	3.00	3.36	3.57	0.755 (0.678–0.831)	0.831 (0.781–0.880)

HLS-14: 14-Item Health Literacy Scale; CI: confidence interval; HL: health literacy; **α**C: Cronbach's alpha; **ωʄ**: McDonald's Omega (internal consistency with BCa bootstrap confidence intervals, 1,000 samples).

Construct equivalence involves demonstrating divergent validity through the relationship of the main scale with other measures. In this study, the HLS-14 instrument was compared with the MOS-SSS questionnaire. As observed in Table 3, divergent validity was evidenced by the absence of significant correlations, as well as the presence of significant correlations of weak magnitude (r<0.300) between the compared dimensions.

**Table 3:** Correlation coefficients between perceived social support (Medical Outcomes Study Social Support Scale) dimensions and health literacy (14-Item Health Literacy Scale) dimensions in adults with mental disorders (n=444).

MOS-SSS	HLS-14							
	Functional literacy		Communicative literacy		Critical literacy		Total	
	r	p	R	p	r	p	r	p-value
Material support	-0.008	0.872	0.046	0.329	-0.074	0.121	-0.011	0.810
Affective support	-0.009	0.843	0.028	0.553	-0.055	0.247	-0.013	0.782
Emotional support	0.059	0.213	0.222**q	0.000	0.104*	0.028	0.163**	0.001
Informational support	0.002	0.959	0.162**	0.001	0.046	0.334	0.085	0.073
Emotional/informational support	0.033	0.492	0.203**	0.000	0.079	0.095	0.131**	0.006
Positive social interaction	0.030	0.533	0.186**	0.000	0.141**	0.003	0.145**	0.002

HLS-14: 14-Item Health Literacy Scale; MOS-SSS: Medical Outcomes Study Social Support Scale; r: factor correlation coefficient.

* Statistically significant correlation at p<0.05.

** Statistically significant correlation at p<0.01.

## DISCUSSION

This cross-sectional study demonstrated that the HLS-14 instrument has valid and reliable psychometric properties for measuring HL in people living with MDs. This is the first Brazilian study employing the HLS-14 in this population. Participants presented low Functional literacy and high Critical and Communicative literacy.

Validating a tool that allows the assessment of HL for this specific population is important, given the considerable prevalence of MDs in Brazil^2,4^ and worldwide^23^, as well as the important relationship between HL and autonomy and treatment adherence^10,11^. Furthermore, most studies on HL of people living with MDs are limited to assessing the Functional dimension, providing only a partial understanding, since HL is currently a multidimensional construct^15^. Thus, the validation of the HLS-14 is important, as it may play a valuable role in identifying impaired dimensions and planning targeted interventions by assessing all three dimensions.

Confirmatory factor analysis demonstrated adequate model fit, consistent with previous validations^16,17^. The RMSEA was 0.061, indicating an acceptable model fit^24^. Based on the goodness-of-fit indicators obtained, it is noteworthy that the evaluation of model fit generally requires that at least three fit indices meet or exceed the recommended thresholds for adequate fit. In this regard, the three-factor model showed a substantial number of satisfactory fit indicators, providing evidence of the high quality of the factor structure tested for the HLS-14.

Reliability was adequate, with an overall Cronbach's alpha of 0.83 (>0.70), consistent with previous studies (0.81–0.82)^16,17^. McDonald's omega was 0.755, confirming internal consistency. Dimension-specific alphas were 0.83 (Functional), 0.90 (Communicative), and 0.77 (Critical), comparable to the literature^16,17^, with slight variation in the Functional domain compared to the Brazilian validation study^17^.

The lowest mean was observed for Functional HL (2.83), whereas Communicative (3.57) and Critical (3.62) dimensions showed similar values. Greater variability was found in the Functional domain. These values suggest that, while the study participants are reasonably good at understanding and using health information, they may face difficulties in more basic aspects, such as reading and initial comprehension of the information. Furthermore, considering that the functional dimension seeks to assess basic reading and writing skills in everyday situations, the low average score in this dimension may be explained by the sample in this study being composed mostly of people with low education level (between incomplete and complete primary education) and low socioeconomic status, factors associated with poorer HL^12^. These findings also corroborate the results of Gheno et al., which also found an influence of skin color and age on HL^21^.

All factor loadings were >0.400, demonstrating the explanatory strength of the items and supporting their retention in the instrument. However, unlike previous studies, a borderline factor loading was observed for item 10 ("I apply the information I receive, through self-assessment, to my lifestyle and daily routine."), although it remained acceptable and relevant. This lower factor loading, compared with the other items, may be explained by the greater variability in responses, which could be related to the complexity of the statement and the resulting difficulty in its comprehension. Furthermore, cultural particularities and the clinical context of the participants should be taken into account, given that the study population comprised individuals with different mental disorder diagnoses.

Regarding the correlation among the three dimensions of HL, it was verified a high correlation between Communicative and Critical dimensions, and a low correlation between these and the Functional dimension, as previously reported^16,17^, reinforcing the multidimensional nature of HL. This finding suggests that basic information collected from the Functional dimension cannot replace data from the Communicative and Critical dimensions, which assess more advanced knowledge. Therefore, it reinforces the importance of using an instrument that not only assesses the Functional dimension but also takes into account the multidimensionality of literacy.

Divergent validity was confirmed by the absence of significant correlations or weak correlations (r<0.300) with the MOS-SSS^22^. Furthermore, comparison of this finding with previous studies was not possible, as no studies assessing divergent validity between these instruments were identified in the literature.

To our knowledge, this is the first study to validate the HLS-14 for use among people living with MDs. Strengths include the use of a previously validated instrument^17^, brief administration, and a large sample. However, some limitations should be considered. The questionnaire was administered by examiners; therefore, the HLS-14 items were subject to explanations and clarifications provided to participants, which may have influenced their understanding of the items and, consequently, their responses. The sample consisted of individuals receiving care at a health service, characterizing a convenience sample that was not selected using statistical sampling procedures. In addition, it included patients with different MD diagnoses, which may have influenced the psychometric performance of the HLS-14. Lastly, the cross-sectional design did not allow the assessment of test-retest reliability, preventing the evaluation of the tool's reproducibility over time. Therefore, future studies using random and representative population-based samples, as well as longitudinal designs, are recommended to confirm the findings of the present study.

The findings of the present study show that the HLS-14 maintained adequate psychometric properties in individuals with MDs, which is of extreme importance since this population often faces challenges in accessing, understanding, and using health information^25^. Reliable assessment of HL may help find needs support care that focuses on the patient and direct efforts to improve treatment adherence, self-care and overall health^15^. Thus, the HLS-14 can play an important role in identifying needs and improving health outcomes for individuals living with MDs, offering an assessment not only of Functional HL, but of the Communicative and Critical dimensions as well.

## CONCLUSION

The HLS-14 showed evidence of validity and acceptable reliability for assessing HL in Brazilian adults and older adults with severe and persistent mental disorders. The instrument demonstrated acceptable internal consistency, adequate fit to the proposed three-factor structure, and evidence of divergent validity, supporting its potential use in this population.

## Data Availability

The datasets generated and/or analyzed during the current study are available from the corresponding author upon reasonable request.

## References

[1] World Health Organization (2020). International Classification of Diseases 11th Revision (ICD-11).

[2] Brunoni AR, Suen PJC, Bacchi PS, Razza LB, Klein I, Santos LA (2023). Prevalence and risk factors of psychiatric symptoms and diagnoses before and during the COVID-19 pandemic: findings from the ELSA-Brasil COVID-19 mental health cohort. Psychol Med.

[3] Lopes CS (2020). How is Brazilian's mental health? The importance of birth cohorts for better understanding the problem. Cad Saude Publica.

[4] Barbosa CG, Meira PRM, Nery JS, Gondim BB (2020). Epidemiological profile of the users of a Psychosocial Care Center. SMAD, Rev Eletrônica Saúde Mental Álcool Drog.

[5] Karambelas GJ, Filia K, Byrne LK, Allott KA, Jayasinghe A, Cotton SM (2022). A systematic review comparing caregiver burden and psychological functioning in caregivers of individuals with schizophrenia spectrum disorders and bipolar disorders. BMC Psychiatry.

[6] Alemu WG, Due C, Muir-Cochrane E, Mwanri L, Azale T, Ziersch A (2024). Quality of life among people living with mental illness and predictors in Africa: a systematic review and meta-analysis. Qual Life Res.

[7] Arias D, Saxena S, Verguet S (2022). Quantifying the global burden of mental disorders and their economic value. EClinicalMedicine.

[8] Sousa RA (2022). Literacia para a saúde: habilidades para lidar com as informações sobre saúde podem ajudar a construir novos caminhos na saúde pública. RECIIS.

[9] Lor M, Koleck TA, Bakken S, Yoon S, Dunn Navarra AM (2019). Association between health literacy and medication adherence among hispanics with hypertension. J Racial Ethn Health Disparities.

[10] Rossato RO, Rocha SHDN (2020). A importância da adesão ao tratamento e o letramento em saúde: uma revisão de literatura. Braz J Health Rev.

[11] Suhail M, Saeed H, Saleem Z, Younas S, Hashmi FK, Rasool F (2021). Association of health literacy and medication adherence with health-related quality of life (HRQoL) in patients with ischemic heart disease. Health Qual Life Outcomes.

[12] Huang CH, Talley PC, Lin CW, Huang RY, Liu IT, Chiang IH (2020). Factors associated with low health literacy among community-dwelling women in Taiwan. Women Health.

[13] Shahid R, Shoker M, Chu LM, Frehlick R, Ward H, Pahwa P (2022). Impact of low health literacy on patients’ health outcomes: a multicenter cohort study. BMC Health Serv Res.

[14] Nutbeam D (2000). Health literacy as a public health goal: a challenge for contemporary health education and communication strategies into the 21^st^ century. Health Promot Int.

[15] Degan TJ, Kelly PJ, Robinson LD, Deane FP, Smith AM (2021). Health literacy of people living with mental illness or substance use disorders: a systematic review. Early Interv Psychiatry.

[16] Suka M, Odajima T, Kasai M, Igarashi A, Ishikawa H, Kusama M (2013). The 14-item health literacy scale for Japanese adults (HLS-14). Environ Health Prev Med.

[17] Batista MJ, Marques ACP, Silva MF, Alencar GP, Sousa MDLR (2020). Translation, cross-cultural adaptation and psychometric evaluation of Brazilian Portuguese version of the 14-item Health Literacy Scale. Cien Saude Colet.

[18] Peres PCN, Pessoa KR, Bernuci MP, Massuda EM, Yamaguchi MU (2017). Literacia em saúde no brasil: estudo cienciométrico. Encicl Biosfera.

[19] Moreira WC, Sousa AR, Cardoso RDSS, Queiroz AM, Oliveira MAF, Sequeira CADC (2022). COVID-19 in Brazil: are there any differences in Mental Health Literacy between young and aged men?. Rev Lat Am Enfermagem.

[20] Pavão ALB, Werneck GL (2021). Health literacy in low- and middle-income countries: a systematic review. Cien Saude Colet.

[21] Gheno EER, Rodrigues FO, Kunkel GK, Nogaro A, Anastácio ZFC, Kolankiewicz ACB (2024). Association between sociodemographic variables and health literacy levels among individuals with mental disorders in Brazil. J Health Literacy.

[22] Griep RH, Chor D, Faerstein E, Werneck GL, Lopes CS (2005). Construct validity of the Medical Outcomes Study's social support scale adapted to Portuguese in the Pró-Saúde Study. Cad Saude Publica.

[23] Wu Y, Wang L, Tao M, Cao H, Yuan H, Ye M (2023). Changing trends in the global burden of mental disorders from 1990 to 2019 and predicted levels in 25 years. Epidemiol Psychiatr Sci.

[24] Kline RB (2023). Principles and practice of structural equation modeling.

[25] Mantell PK, Baumeister A, Christ H, Ruhrmann S, Woopen C (2020). Peculiarities of health literacy in people with mental disorders: a cross-sectional study. Int J Soc Psychiatry.

